# Progress and challenges of disaster health management in China: a scoping review

**DOI:** 10.3402/gha.v7.24986

**Published:** 2014-09-10

**Authors:** Shuang Zhong, Michele Clark, Xiang-Yu Hou, Yuli Zang, Gerard FitzGerald

**Affiliations:** 1 Center for Emergency and Disaster Management, School of Public Health and Social Work, Institute of Health and Biomedical Innovation, Queensland University of Technology, Brisbane, Australia; 2 Center for Health Management and Policy, Shandong University, Jinan, China; 3 School of Clinical Sciences, Queensland University of Technology, Brisbane, Australia; 4 School of Nursing, Shandong University, Jinan, China

**Keywords:** China, disaster management, disaster response, health system, health policy

## Abstract

**Background:**

Despite the importance of an effective health system response to various disasters, relevant research is still in its infancy, especially in middle- and low-income countries.

**Objective:**

This paper provides an overview of the status of disaster health management in China, with its aim to promote the effectiveness of the health response for reducing disaster-related mortality and morbidity.

**Design:**

A scoping review method was used to address the recent progress of and challenges to disaster health management in China. Major health electronic databases were searched to identify English and Chinese literature that were relevant to the research aims.

**Results:**

The review found that since 2003 considerable progress has been achieved in the health disaster response system in China. However, there remain challenges that hinder effective health disaster responses, including low standards of disaster-resistant infrastructure safety, the lack of specific disaster plans, poor emergency coordination between hospitals, lack of portable diagnostic equipment and underdeveloped triage skills, surge capacity, and psychological interventions. Additional challenges include the fragmentation of the emergency health service system, a lack of specific legislation for emergencies, disparities in the distribution of funding, and inadequate cost-effective considerations for disaster rescue.

**Conclusions:**

One solution identified to address these challenges appears to be through corresponding policy strategies at multiple levels (e.g. community, hospital, and healthcare system level).

Disaster health management is fast becoming a unique specialty around the world, with its governing theories and principles (1). Essential phases of disaster management to improve the effectiveness of the disaster health response have made use of the ‘PPRR’ continuum of prevention and mitigation (P), preparation and planning (P), response and relief (R), and recovery (R) (2, 3). The ultimate goal of disaster health management is to reduce the impact of disasters on human health and wellbeing by providing urgent health interventions and ongoing health care during and after disasters (4, 5). During a disaster, the healthcare system becomes a high profile element, critical to the immediate health response and recovery phase. The system itself can be impacted directly by the consequences of the disaster while at the same time being expected to have the capacity to respond to the sudden increase in the demand associated with the disasters (5, 6). The system, because it provides continuous health care, can be viewed as community infrastructure essential to the life-preserving front-line response (7).

Most of the extant research has occurred in high-income countries, such as the United States of America and has focused on the health system's disaster management (disaster health management), or the capability to supply medical services during disasters (1, 5, 7, 8). However, there is little available information from low- and middle-income countries (4). China, one such country, has been severely affected by multiple kinds of disasters including natural and manmade disasters and pandemics of infectious diseases (9, 10). To date, disaster management research into the health system in mainland China is in its infancy. While many studies in the Chinese language have been published in national medical journals, they often lack scientific rigor (e.g. inappropriate study design, and the lack of empirical data).

Only a small number of investigators have published their studies in peer-reviewed international journals. Moreover, few studies have evaluated the effectiveness of the current disaster arrangements. Thus, there is an opportunity for researchers to share China's experience with international communities about the impact that disasters have on the health response systems. Hence, it is essential to identify the full extent of the challenges that confront China in order to gain an understanding of those areas that require policy improvement and to assist in identifying strategies into the future. These challenges can also be used to benchmark with some high-income countries (e.g. the United States) to identify any gaps and priorities for improving disaster health management strategies.

This paper aims to provide an overview of the status of disaster health management in China. It has several objectives: 1) to identify the progress or current status of disaster management of the healthcare system in China; 2) to identify current challenges; 3) to discuss future strategies to overcome these challenges; and 4) to identify future research directions. The ‘PPRR’ disaster management continuum can be used to identify the progress and the challenges within each management phase. Then corresponding strategies are proposed to promote the overall effectiveness of the health system response during and after major disasters and to reduce disaster-related mortality and morbidity by providing continuous healthcare.

## Methods

The aim of this review was to provide an overview of the extent of the challenges of disaster management rather than to undertake an in-depth assessment of individual studies. For this reason, we conducted a scoping review rather than a systematic review. The aim of the scoping review was to rapidly map the key concepts underpinning the research area using the main sources and types of evidence available (11). A scoping review can be used to address topics that are too broad for a systematic review, or have not been previously reviewed comprehensively (11). Although we did not use meta-analysis (an approach commonly used for systematic reviews), systematic review methods were used where possible to minimize bias in the identification and inclusion of the studies (12). The ‘PPRR’ continuum offered a preliminary framework which was used as a guide to identify the progress and challenges of disaster health management.

The major health electronic databases including ProQuest, PubMed, EBSCO, ScienceDirect, Web of Science, and the Chinese Biomedical Literature Database were searched to identify publications such as public reports and peer-reviewed journal articles, which were relevant to the research aims. The search terms and their logical relation (e.g. AND) were: ‘disaster OR emergency’ AND ‘medical OR health OR hospital’ AND ‘management OR preparedness OR response’. Additional references were identified through an examination of the references from recent pertinent publications (snowballing) and through scrutiny of the contents pages of highly relevant journals for the previous 2 years.

The research inclusion criteria were: 1) journal articles, governmental and institutional reports written in English or Chinese in the past two decades; 2) studies comprising relevant evaluations of the status or description of the progress and challenges of disaster management (i.e. disaster prevention, preparedness, responsiveness, and recovery) of the healthcare system in China; and 3) other jurisdictions that had direct relevance to disaster health management in China (e.g. disaster healthcare management, disaster medical responses, emergency medical care, and emergency healthcare systems).

The research exclusion criteria were: 1) studies that only focused on disaster management of specific healthcare systems of other countries, without any implications to China; and 2) studies with no detailed evaluations or descriptions that could assist in informing the identification or description of the progress and challenges of disaster health management in China.

The article titles were scanned by two reviewers independently for relevance to the research aims; then the abstracts were appraised for relevance, significance, and utility. Next, the full text format was retrieved for the remaining publications and analyzed in relation to their contributions to two areas: the identification of the main progress and challenges of disaster health management in China and the description of such progress and challenges.

Initially, a total of 362 potentially eligible publications were retrieved. Of these, 231 were excluded through the screening of their titles and abstracts. After scanning the full text of the remaining 131 publications, 37 relevant publications were identified as potentially relevant to the current study; they included governmental and institutional reports and journal articles written in English or Chinese. After an analysis of these publications, all 37 were assessed as relevant to the study's aims, and thus were included in the review.

## Results

### Progress of disaster health management in China

Over the past decade, China has witnessed a series of major disasters. As a consequence the ability of the health system to respond to disasters has improved significantly. Many of the resultant changes that have occurred stem from the lessons learned from these disasters and were implemented in an attempt to better respond to disasters in the future. First, in response to the 2003 SARS crisis, the government acted to improve the prevention and management of infectious diseases. These initiatives included the establishment of a national infectious disease surveillance system and independent infectious disease hospitals; improved isolation facilities in Emergency Departments (ED); the upgrading of the isolation wards; improved training and monitoring of hospital staff in infection-control procedures; and improved compliance with the use of personal protection equipment (13–15).

A national integrated emergency response system has also been developed and promoted. China's National Committee for Disaster Reduction (NCDR) was established in 2005 as the state inter-agency coordination body. It comprises 34 ministries and departments, as well as military agencies and social groups (10). The integrated system seeks to ensure the effective management of resources and rescue personnel from different facilities throughout China (16).

In addition, there has been an integration of military medical resources into the disaster management system. The army hospitals have advantages that include: intrinsic infrastructure, well-trained staff, modern equipment, and communications and transportation systems (17, 18). In 2010, China began establishing 22 medical emergency teams across the country to respond to different disasters (19). Many of these teams can be deployed from military hospitals. Army hospitals are fully equipped with portable medical equipment and independent living supplies, so that they do not need to use local supplies (19, 20). They provide healthcare services by establishing temporary field hospitals, accepting and transferring patients, or providing expert rescue teams onsite (19, 20).

Despite this progress, there remain challenges that hinder efficient disaster health management in China. Such challenges have been caused mainly because China is still in the early stages of health disaster management development (14). These challenges were identified and extracted from the literature and described in detail below.

### Challenges of disaster health management in China

#### Health infrastructure safety

The 2005 World Conference on Disaster Reduction endorsed a number of policies to ensure that ‘all new hospitals are built with a level of resilience that strengthens their capacity to remain functional in disaster situations’ (21, 22). Disaster-resilient infrastructure is a primary guarantee for health care organizations to maintain their functions during disasters; they achieve this outcome through their ability to resist and absorb disaster impacts on physical facilities. Resilient infrastructure includes not only physical strength but also back-up for the systems. However, no standard has been endorsed or enforced to ensure that healthcare facilities can resist natural disasters. In addition, back-up systems (e.g. electricity, water, and communication) were not fully considered when many hospitals were being built. For example the health facilities in the earthquake-prone areas of western and rural China rarely comply with the standards of construction, nor are their back-up systems required to resist natural disasters (23). The 2008 Sichuan earthquake caused the collapse of 67.5% of healthcare buildings in the worst affected areas (24). As a consequence, a large proportion of the county hospitals were destroyed or lost their critical systems. It is also noted that a number of the township hospitals and village clinics required temporary facilities to support their ongoing roles (25). Thus, in China, the low standard of disaster-resilient infrastructure is the first challenge to efficient disaster response (6).

#### Disaster preparedness

An effective disaster response can be achieved only through sufficient preparedness before the occurrence of any disaster (26). However, several studies based on hospital evaluation surveys have revealed that China is still in the early stages of developing hospital emergency preparedness (13, 14, 27). In many provinces, hospitals were found not to have specific disaster plans for natural disasters that have a low frequency of occurrence (e.g. earthquakes and floods), novel infectious diseases, or terrorism attacks (particularly biological, nuclear, and radiation attacks) (13, 14, 28). Moreover, the health facilities in many regions have a low level of essential preparedness in relation to disaster vulnerability analysis, disaster stockpiles, coordination with other institutions, emergency training in disaster first-aid, rescue, and the use of specialized supplies (13, 14, 27, 28). Western and rural area hospitals are even less prepared, having lower proportions of these essential preparedness aspects (24, 25, 29).

The availability of medical devices and equipment, especially the miniaturization and portability of medical devices, are crucial for the initial disaster medical response, as well as for onsite rescue (30). For instance, portable kidney Doppler ultrasonography devices are effective for initial diagnoses and triage during mass casualty disasters (30). However, there are still inadequate portable medical devices in China. As happened during the 2008 Wenchuan earthquake, an enormous amount of hospital equipment was unavailable in the hardest hit areas, with the larger equipment not being appropriate for onsite triage and treatment. Further, during this event, most of the rescue teams were not prepared; they did not have portable radiography machines or ultrasonography facilities (23). Such inadequacy may impede the ability of the rescue teams to provide first-line medical treatment during future natural disasters (31).

Emergency supplies are also an essential component of hospital disaster preparedness (28). While one study recently found that more than 75% of tertiary hospitals in the Shandong Province had stockpiles of emergency supplies (e.g. medicine, food, water, stretcher, and tourniquet), only a small number (29.3%) of hospitals had signed a memorandum of understanding with other regional hospitals to share these supplies during disasters (28).

#### Disaster medical response capability

A rapid and effective medical response by the local health services can be seen as the front-line of rescue efforts. This response is critical for facilitating the process of field triage, transport, and transfer (32). Consideration of these factors assists the rational allocation of healthcare resources during disasters. Currently, several crucial aspects for an effective health response are inadequate. The establishment of the triage criteria, based on the severity of the disaster and the availability of the health resources, is central to improving healthcare capacity during disasters (24, 33). A simple triage and rapid treatment (START) method was established after the Wenchuan earthquake to facilitate site triage and injury classification (33). However, no standard triage procedure or guidelines have been fully adopted in China. Instead, most hospitals have adopted disaster triage procedures from the general procedures used in ED (31, 33).

The skills of the emergency staff in disaster management such as disaster triage skills were also found to be wanting, mainly due to the lack of targeted and appropriate disaster education and training programs. For example, the literature revealed that a large proportion of doctors had not received any formal training in triage, effectively relying on their own judgment which might cause bias, a delay in treatment or even waste scarce resources (31, 33). in addition, medical students also failed to receive appropriate disaster training. They acquire their training and skills in the inpatient wards of large tertiary care hospitals in urban areas where the emphasis is on making the right diagnosis rather than on the principles of triage and emergency management (34).

To be effective, it is essential that hospitals surge their patient-care capacity in a short period of time after a disaster (e.g. within 24–72 hours) (35–37). However, Chinese disaster surge capacity still lags behind other countries such as the United States (28). As revealed by previous research, most secondary and tertiary hospitals in Beijing acknowledge they have insufficient surge (extra) beds to meet the demands during disasters, such as an infectious disease epidemic. The surge beds accounted for only 8.5% of all the fixed beds after the SARS crisis in 2004 (13). In 2012, only 65.9% of the tertiary hospitals in Shandong Province were able to surge patient-care beds, with the total surge capacity being 12.52%, within 24 hours (28). Two reasons were identified for this low surge capacity. First, there was a lack of a hospital surging plan that used flexible surging strategies during disasters (35, 36, 38–40). For instance, in 2012, only 53.7% of the tertiary hospitals in the Shandong Province had surging plans; only 36.6% of the hospitals adopted a variety of flexible procedures for surging their beds (e.g. through the early discharge of patients, the cancellation of elective admissions, or the transfer of patients). Second, the health system was already under increased pressure from the growing daily demand (9, 29, 41). This human resource shortage compounded the limited surge capacity of the hospitals during disasters. As noted earlier, during the 2008 earthquake, the local healthcare workers were overwhelmed by the large number and the severity of casualties (19).

#### Disaster recovery

When the acute phase of a disaster ends, the challenge moves to sustaining the long-term rehabilitation of the population, particularly those with disabilities and chronic diseases (26, 42). The psychological intervention guidelines for public emergencies were sued by the Ministry of Health. The guidelines stipulated two phases for psychological interventions. The first phase, the acute phase, occurs when general psychological counselling is used to reduce the incidence of posttraumatic stress disorder. The second phase, the chronic phase, occurs when psychological interventions are focused on issues associated with depression (23). However, to date, few programs exist for the evaluation and identification of psychological problems (especially during the chronic phase) of the population in the disaster areas. As a result, the targeted interventions remain inadequate for the treatment of large numbers of victims with psychological problems (e.g. post-injury stress disease), or for victims with the potential for psychological problems that arise during disasters (43).

Two factors underpin this inadequacy. First, psychological problems have become common especially during natural disasters and infectious disease outbreaks and these affect both the victims and the rescuers (43). However, psychological problems have not received the same emphasis that physical illnesses and injuries receive.

Second, the local medical staff have not been well-trained in managing severe psychological effects, even in disaster prone areas (25, 43). Further, there has been a nationwide shortage of senior experienced doctors and mental health professionals, which contributes to the lack of sensitivity to patients’ psychological needs and impedes the supply of post disaster psychosocial interventions (24, 43). This workforce shortage became apparent during the Wenchuan earthquake. In essence, there were insufficient professionals in the local area and they could not be dispatched at short notice to respond to the psychosocial problems (24).

#### Supporting systems

Several supporting systems were found to be wanting in terms of aiding the delivery of disaster relief emergency services, namely: the fragmentation of emergency health service systems, the lack of specific emergency legislation, the disparities in funding distribution, and inadequate cost-effective considerations. These systems are discussed below.

*Fragmentation of emergency health service system*. The pre-hospital emergency service is arguably the least developed aspect of the emergency medical service system. There are large variations in the structure of the pre-hospital emergency service across China (18). Some large cities have independent pre-hospital emergency services, while others rely on hospitals (18). Hence, the roles of hospital EDs and emergency service centers overlap in some large cities such as Beijing and Shenyang (18). Moreover, there is no official guideline, protocol, or legal standard for patient management and transfer between these two sectors. During disasters, the independence of these two sectors can lead to inefficiencies and the waste of valuable resources (18, 29). In most regions, pre-hospital emergency services lack effective cooperation with the fire and police departments. A lack of cooperation may result in the loss of precious rescue time for advanced pre-hospital medical care.

*Lack of specific emergency legislation*. In China, the legal foundation for disaster management has been established through the ‘Act on Tackling Emergency Affairs (2007)’ ( 44, 45). However the document is not specific enough to be implemented in the local area. In addition, there are numerous legal obstacles that hinder appropriate disaster health management.

First, there is a lack of a guaranteed reimbursement to the disaster healthcare services; this lack of reimbursement may encourage perverse financial disincentives. This situation may effectively discourage hospitals from becoming involved in disaster preparedness (45). Without legal guarantees, few insurance companies will accept insurance for health staff working in the disaster areas (46). In addition, there is the need to have similar hospital command and control systems across hospitals in all responding sites, to ensure the maximum efficiency of mutual aid. However, to date, there is no such overarching command and control guideline to assist the different areas of China. This lack of legal clarification may impede the formulation of an integrated response system for disaster command, control and cooperation (47). Also, the responsibility and authority of the different levels of government, the army facilities, and the non-governmental organizations are not clearly defined within the law. For non-profit organizations this vagueness may cause chaos during disasters (23). For example, the 2013 Lushan earthquake highlighted the difficulties that can arise in the absence of a legal system to recruit and coordinate the volunteers. In that instance, the individual volunteers and unauthorized organizations entering the disaster area created road congestion and inadvertently, unnecessary casualties (19). Further, without the legal enforcement to release details to the public about the use of donations, embezzlement of some of the donations for the Wenchuan earthquake occurred. This event caused a credibility crisis for the public in regard to the donations given to the government-organized NGOs (Non-Governmental Organizations) (19).

*Disparities in funding distribution*. The funding gaps and the disparities in the distribution of funds present major challenges for healthcare organizations providing medical care during a disaster response (47). The first disparity occurs between the funding of urban and rural areas. For example, healthcare resources, modern healthcare facilities, and physicians are concentrated mainly in the urban areas while the rural areas are less well resourced (4, 29). There is also a tension between the allocation of resources for the immediate day-to-day needs and for disaster preparedness. In addition, investments that are put toward the improvement of emergency preparedness may compromise other more urgent programs such as primary healthcare in rural and western areas (4). In addition, most funding was used for the reimbursement of actual expenses after a disaster. The 2008 government finance report indicates that most government funding was used for the Wenchuan earthquake rescue (the central government invested 38.4 billion RMB, about US$6.28 billion), while the earthquake relief and preparedness funding amounted to 2.4 billion RMB (about US$0.4 billion). Finally, since the health system reforms were introduced in the 1980s, healthcare organizations have turned their attention to revenue-generating services (9). As a result, hospitals and professionals can be paid significantly more for their clinical services than their disaster-related work (9). Thus, without sufficient financial allocations the motivation of hospitals to improve their disaster preparedness is likely to remain low.

*Inadequate cost-effective considerations*. The cost-effectiveness of disaster management is easily neglected especially during catastrophic disasters. When the disaster occurs the decisions about who and what to send to the disaster zone might be determined by dogma, rather than by scientific analysis (25). For instance, during natural disasters, a large number of search and rescue teams are dispatched to the disaster zones and part of the purpose of their role is moral inspiration (25). However, if there is an oversupply of search and rescue teams, the influx of too many teams may become a burden on the limited supplies for the victims, such as food, shelter, sanitation, and healthcare services (25). Occasionally, outside aid agencies have been known to rush manpower, equipment, and supplies to a disaster area regardless of the local requirements, and without coordination with the other local organizations’ plans and resources; this situation may lead to the waste of resources and result in low efficiency (19, 25). For instance, during a disaster, supplies are delivered from the outside (such as clothes and foods); these donations can be useless as they are inappropriate for the current situation. Moreover, receiving these donations may cause a waste of valuable warehouse space and manpower. Also, because the source is unknown, the donated items may need to be sterilized properly, which uses even more resources (25).

## Discussion

Several contributions have been made by this review to the broader body of knowledge. First, the review identified comprehensively the progress and challenges of disaster health management in China; this outcome was achieved through the extraction of relevant information from the literature, in both the English and Chinese languages. Indeed, the topic was found to be poorly covered. Importantly, the review offered the opportunity to assess and evaluate the current status of disaster health management. It also provided a foundation for further in-depth analysis of the individual challenges and the progress achieved. Second, some challenges involved the internal aspects of the health system while others arose from the external environment. The ‘PPRR’ disaster management continuum integrates the internal challenges; it also offers a preliminary framework which can be used to highlight the progress and the weaknesses of each management phase. Further, this continuum can be used to develop a proposed multi-strategic approach to address corresponding challenges with a view to enhancing disaster management in the future.

Most of the information that informed this scoping review of disaster health management in China was in the format of qualitative descriptions and analyses. Because the topic has been poorly researched, few quantitative studies with empirical data were available for appraisal. Further, these quantitative studies had limitations including the short length of the study period and the examination of samples of hospitals in a small number of locations or regions, for example, Beijing, Sichuan, and Shandong. Because of the limited amount of research in the literature, and in particular empirical research, the current study used scoping review methods to locate research materials that could then be used for a systematic review. Despite the aforementioned limitations, the methods were appropriate for a policy analysis topic such as the current study which sought to identify the current status of and challenges to health disaster management in China. The limitations identified in the literature in terms of need for more rigorous research designs and information on health services in more areas of China provide considerable potential for future research.

### Policy implications to overcome these challenges

The current study revealed that the healthcare facilities’ preparedness for disasters was under challenge by the vulnerability of the physical infrastructure, inadequate disaster plans, and disaster resourcing, and funding considerations. To enhance local disaster preparedness, multiple strategies need to be adopted. First, the local prioritized hazards need to be evaluated, while strict structural standards need to be enforced; these aspects are essential for reducing casualties from disasters (21, 48). For example, in the hardest hit area of the 2013 Lushan earthquake, 96.32% of the public buildings built after the 2008 Wenchuan earthquake were still functional and able to be used. As a result, the new structural standards reduced the impact of the disaster on human health and wellbeing (19). Second, operational disaster plans for healthcare facilities need to be devised, in advance. In addition, they need to take into consideration the communities’ resources, hazards and other unique factors (14, 49). Third, healthcare organizations need to have the capacity to be self-sufficient for the first 48 to 72 hours; this disaster coping capacity is essential as it may take this long for supplies to be delivered from the outside (32). Fourth, non-governmental mutual assistance, as well as a social insurance mechanism, needs to be strengthened to decrease the gap between the governmental funding and the actual costs of the disaster relevant work (49, 50). Finally, funds need to be allocated to local primary healthcare centers and hospitals to assist their roles in providing adequate capability as the first disaster responders (7).

The scoping review revealed that research on surge capacity exists in developed countries, such as the United States; however, its concept and strategies are not fully adopted in the Chinese context. Nevertheless, some international surge strategies would appear to have the potential to be adapted into the Chinese disaster-planning environment. These surge strategies would include: providing staff with onsite accommodation, and the training of non-clinical staff to support the fully trained staff (51, 52), cancelling elective surgery in order to focus on critical care (51), triaging the resources, and providing patients with adjusted standards of healthcare during the period of the disaster (53, 54). Other strategies would include converting non-clinical areas for surging clinical space (prepared in advance, with available power, water, oxygen, equipment and telecommunication) (51, 55, 56); early discharge of stable inpatients or referring them to ancillary healthcare services (e.g. nursing homes and primary healthcare centers) (51, 55, 56); and obtaining cooperative agreements with other facilities and off-site hospitals (38, 51). Such considerations have the potential to assist the country to surge its capacity during times of disaster.

In China, psychological interventions, triage skills, and other disaster management skills are limited by inadequate education in disaster management. Thus, appropriate education or training curricula need to be developed and implemented to address these issues in the long term (49). Such courses need to be available for hospital professionals as part of their ongoing professional development. Systematic and ongoing training of staff in disaster skills and equipment usage should be conducted in high-risk communities. For example, staff should be trained in how to triage a large number of patients and with limited resources. The training programs used in other countries could be implemented but with appropriate modification to fit the Chinese environment, as necessary. The American Medical Association has developed two courses that have this potential, but which are largely directed to the initial triage and management in the field and in the ED, namely the Basic Disaster Life Support course, and the Advanced Disaster Life Support course (20).

Currently, the fragmentation or lack of coordination of the emergency systems in China hinders efficient disaster management. The close coordination of the medical rescue services (e.g. government and non-government, domestic and international) is essential to overcoming this challenge (49). Such cooperative channels should include strengthening effective cooperation from pre-hospital centers, hospital ED, and fire and police departments (41); establishing a unified command and information platform for governmental agencies, national delegations, and NGOs (47, 49); and, finally, strengthening NGOs, particularly with respect to their management of volunteers. A system similar to the US national verification system that would enable or facilitate the quick identification, recruitment and coordination of the medical volunteers is worthy of consideration (5).

### Future research directions

Based on this review, a number of research questions have been proposed with the aim of providing scientific evidence as the basis for disaster health management in China and facilitating policy-making that would overcome future challenges. These questions are listed below:

Q 1: How can the new concept of ‘disaster preparedness’ and ‘surge capacity’ be best implemented to prepare the local health system as the first disaster responder and integrate the health system into the local planning network?

Q 2: Can user-friendly and validated tools be developed to evaluate hospital capability to cope with disasters? In order to evaluate the hospitals’ actual ability to cope with disasters, the relevant validated evaluation tools will need to be tested during disaster simulations to identify the beneficial factors. Hence, the tools can be used to monitor and analyze hospital response performance through the disaster drills.

Q 3: How can the research-to-policy interface be bridged? For example, can context-specific domestic disaster guidelines or plans be formulated to encourage vulnerable healthcare organizations to take adequate actions before or during disasters? Can a minimum number of items be identified that should be stored in ED and which take into account specific regional and geographic needs and resources.

Q 4: How can evidence-based research be used to determine and specify whether the levels of ability are linked to a desirable outcome, and is the outcome regarded as adequate? For example, in terms of surge capacity, the thresholds for the extent of, and rapidity for, surge capacity should be investigated with considerations being given to different regional conditions.

## Conclusions

Effective disaster management of the health system is essential for disaster response. This paper has identified the progress of and challenges to the Chinese health system in providing continuous health care services during disasters. These challenges emanate from both the internal components of the health organizations and the external environment, which can directly or indirectly impede effective disaster health management. Solutions that were identified to address these challenges require corresponding policy strategies at community, hospital and healthcare system levels.
